# Synergistic Damage Behavior of 5052 Aluminum Alloy Under CW–Nanosecond Combined Pulse Laser Irradiation

**DOI:** 10.3390/ma19173589

**Published:** 2026-08-24

**Authors:** Yuehao Cai, Donghan Li, Yuyang Chen, Junyang Xu, Xianshi Jia, Lu Zhang, Kai Li, Zhou Li, Cong Wang

**Affiliations:** 1 State Key Laboratory of Precision Manufacturing for Extreme Service Performance, College of Mechanical and Electrical Engineering, Central South University, Changsha 410083, China; 2 School of Intelligent Manufacturing, Hunan First Normal University, Changsha 410221, China

**Keywords:** 5052 aluminum alloy, combined pulse laser, material removal, plasma

## Abstract

5052 aluminum alloy has been widely used in aerospace, shipbuilding, automotive, and electronic industries due to its low density, high specific strength, and excellent corrosion resistance. Understanding its laser-induced damage behavior under combined continuous-wave (CW) and nanosecond (ns) pulse laser irradiation is essential for optimizing combined laser processing. In this study, the damage behaviors induced by individual CW laser, individual ns pulse laser, and combined pulse laser were systematically investigated using high-speed imaging, infrared thermography, and three-dimensional surface characterization. The results show that the combined pulse laser significantly enhances both damage depth and material removal efficiency compared with single laser irradiation. Although the peak surface temperature remains nearly unchanged under different processing conditions, the crater morphology and penetration depth vary substantially. High-speed imaging reveals that plasma evolution and molten metal ejection dominate the material removal process. Variations in processing parameters significantly modify molten pool dynamics and plasma behavior. In particular, enhanced plasma shielding or excessive energy dissipation reduces the effective laser energy coupling, leading to decreased material removal efficiency. The synergistic interaction among molten pool evolution, plasma expansion, and molten metal ejection governs the final damage morphology. This study provides new insights into the dynamic interaction mechanisms between combined pulse laser and aluminum alloys, offering guidance for parameter optimization in high-precision laser micromachining.

## 1. Introduction

Aluminum alloys have been extensively employed in aerospace, automotive, shipbuilding, electronic packaging, and new energy industries owing to their low density, high specific strength, excellent corrosion resistance, and good machinability [1,2,3,4]. Among them, 5052 aluminum alloy, a typical Al–Mg wrought alloy, exhibits outstanding corrosion resistance, weldability, and formability, making it a key structural material for ship hulls, automotive fuel tanks, and electronic heat-dissipation components [5,6,7]. With the increasing demand for lightweight, high-performance, and highly reliable components in advanced manufacturing, higher requirements have been imposed on the machining accuracy, surface integrity, and service reliability of 5052 aluminum alloy [8,9]. Consequently, high-efficiency and high-precision laser processing technologies have become an important approach for its advanced manufacturing [10,11].

Laser processing has been widely applied in metal cutting, welding, drilling, surface modification, and micromachining because of its non-contact nature, high energy density, excellent processing accuracy, superior flexibility, and ease of automation [12,13,14,15]. However, the laser processing of aluminum alloys remains challenging because of their high laser reflectivity, high thermal conductivity, and relatively low melting point [16,17]. These intrinsic properties often result in low laser energy coupling efficiency, unstable molten pool dynamics, severe molten material spattering, and difficulty in controlling the processing quality [18,19]. Therefore, a comprehensive understanding of the interaction mechanisms between laser irradiation and aluminum alloys is essential for improving processing performance and ensuring manufacturing quality [20,21]. Laser-induced damage behavior represents one of the fundamental scientific issues in laser–material interactions, directly affecting material removal efficiency, machining quality, and the service performance of engineering components [22,23]. Nevertheless, the interaction mechanisms differ significantly among various laser sources. CW laser provides continuous thermal energy input and promotes the formation of a stable molten pool. Previous studies by Zhang et al. demonstrated that thermal accumulation during CW irradiation plays a dominant role in determining the heat-affected zone and molten pool evolution [24]. Other investigations further revealed that CW-induced thermal effects are closely related to melt flow behavior and processing stability [25]. In contrast, ns pulse lasers possess high peak power, enabling rapid melting, vaporization, and plasma generation within an extremely short duration. Li et al. reported that transient energy deposition during ns laser irradiation induces intense evaporation and plasma formation, resulting in typical pulsed laser ablation characteristics [26]. Further studies have shown that plasma evolution and recoil pressure significantly influence material removal behavior under ns laser irradiation [27,28]. Damage morphology, penetration depth, molten width, material removal volume, and removal efficiency are widely recognized as key indicators for evaluating laser processing performance, while revealing the underlying damage evolution mechanisms provides the theoretical basis for process optimization and precise quality control [29].

At present, CW laser and ns pulse laser are the two most widely employed laser sources for metallic material processing. CW laser continuously delivers thermal energy to the workpiece, forming a stable molten pool and therefore is well suited for laser welding, laser cladding, and surface modification [30,31]. However, the continuous heat input inevitably leads to a relatively large heat-affected zone, molten pool collapse, and thermal deformation [32]. In comparison, ns pulse laser offers significantly higher peak power, allowing rapid melting, vaporization, and plasma formation with higher machining precision and a smaller heat-affected zone. Nevertheless, their material removal efficiency is limited by the relatively low energy of a single pulse and is readily influenced by plasma plumes and molten particle ejection [33,34].

In recent years, extensive studies have been conducted on molten pool evolution, temperature field distribution, plasma formation, and material removal behavior under CW or ns laser irradiation. Previous studies have demonstrated that the recoil pressure generated by laser-induced vaporization drives vigorous molten metal flow, while the Marangoni effect, surface tension gradients, and thermocapillary convection collectively govern molten pool evolution and ultimately determine the processing quality [35,36,37]. Meanwhile, the rapid development of advanced diagnostic techniques, including high-speed imaging, infrared thermography, and numerical simulation, has provided powerful tools for revealing the transient physical processes involved in laser processing [38,39].

Recent studies on combined pulse laser processing have primarily focused on improving processing efficiency, regulating molten pool stability, and optimizing machining quality [40]. Masinelli et al. further improved processing efficiency through a customized sequential multi-ring laser drilling approach [41]. Shmatko et al. investigated the influence of argon (Ar) and nitrogen (N_2_) shielding gases on molten pool stability by drawing on knowledge from laser welding and systematically evaluated the effects of different processing parameters, demonstrating that appropriate gas selection effectively stabilizes the molten pool, suppresses defect formation, and improves the mechanical properties of metallic components [42]. Khan et al. optimized the molten pool geometry during high-speed laser welding of thin-gauge automotive steels by adjusting the laser incident angle, thereby enhancing process stability and reducing welding defects without requiring additional consumables or complex process configurations [43].

In the field of combined pulse laser-induced damage, Wu et al. investigated the cutting process and damage mechanisms of thick CFRP laminates using waterjet-guided laser machining and, for the first time, identified three characteristic damage mechanisms, namely exposure, spalling, and fiber pull-out, providing valuable guidance for precision machining of thick CFRPs [44]. Li et al. proposed a hybrid laser ablation–drilling technique for micro-hole fabrication in woven carbon fiber-reinforced silicon carbide (Cf/SiC) composites, which significantly improved machining performance while reducing tool wear [45]. Li et al. developed an innovative laser shock cleaning and strengthening process (LSCSP), in which the synergistic effects of high-energy pulse laser irradiation and a liquid confinement layer achieved efficient coating removal and substrate strengthening through thermal evaporation, plasma shock waves, substrate elastic–plastic deformation and hydrodynamic erosion [46].

Although considerable progress has been made in understanding molten pool evolution, plasma dynamics, and material removal mechanisms during combined pulse laser processing, the dynamic damage behavior of 5052 aluminum alloy under the combined irradiation of CW and nanosecond ns laser remains insufficiently understood. In particular, the complex coupling among surface temperature evolution, plasma dynamics, molten pool flow, and molten metal ejection during combined pulse laser irradiation has not yet been systematically clarified. Consequently, the mechanisms by which these coupled phenomena govern the final damage depth, crater morphology, and material removal efficiency remain unclear.

Therefore, in this work, systematic damage experiments were carried out on 5052 aluminum alloy under individual CW laser irradiation, individual ns pulse laser irradiation, and combined pulse laser irradiation. High-speed imaging, infrared thermography, three-dimensional surface morphology characterization, and cross-sectional profile analysis were employed to investigate the evolution of surface temperature, plasma behavior, molten metal ejection, and the dynamic damage process during combined laser irradiation. Particular attention was paid to elucidating the effects of CW laser power and ns pulse energy on the damage behavior of the combined laser system, as well as the synergistic mechanisms among molten pool evolution, plasma dynamics, and material removal. The results provide both theoretical guidance and experimental support for optimizing combined laser processing technologies and advancing the precision manufacturing of aluminum alloys.

## 2. Materials and Methods

**Figure 1:**
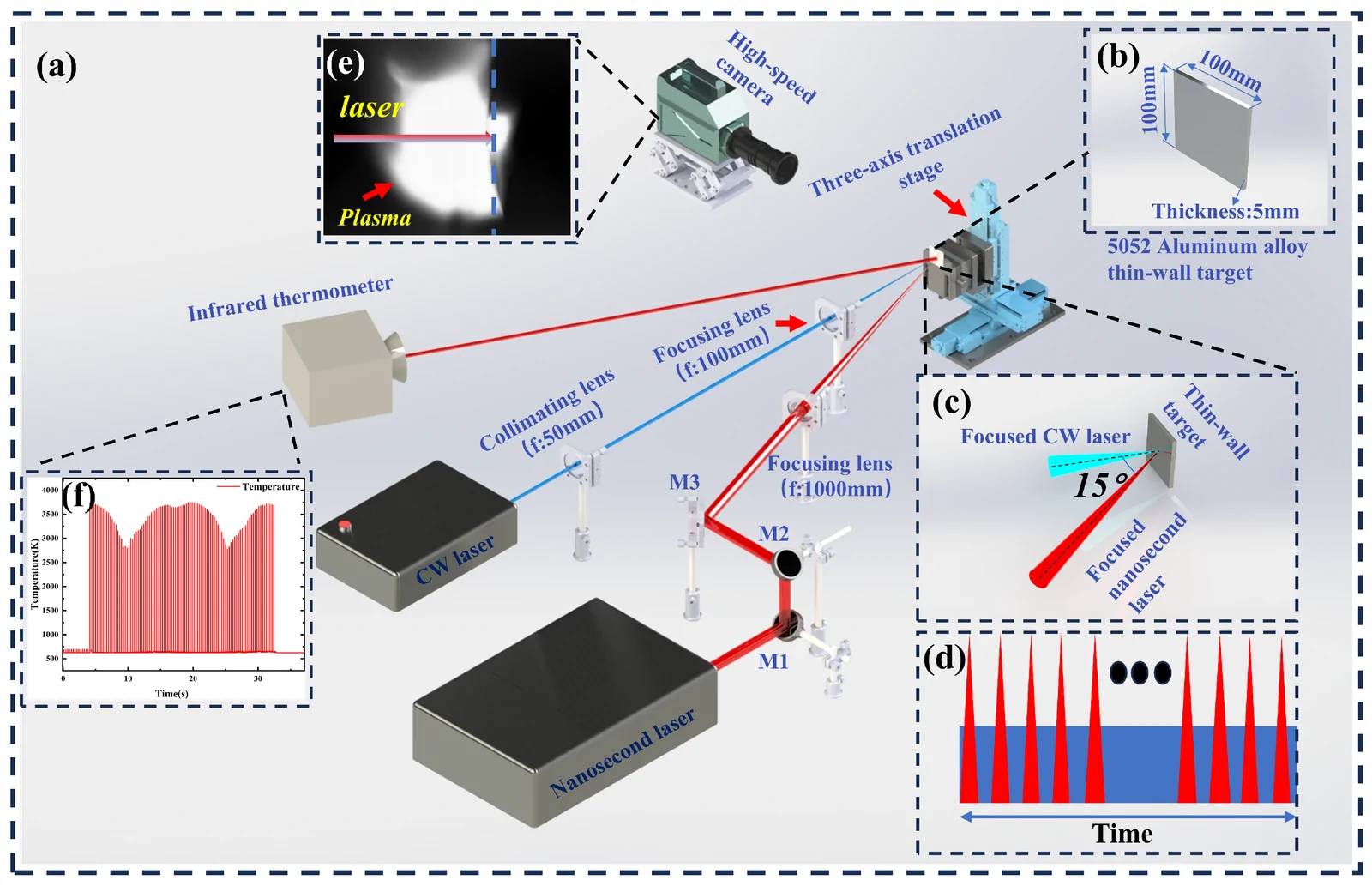
Experimental setup and diagnostic methods: (**a**) schematic of the experimental system; (**b**) 5052 aluminum alloy thin-wall target; (**c**) schematic of combined pulse laser irradiation on the target material; (**d**) pulse timing sequence of the combined pulse laser; (**e**) representative high-speed images; (**f**) transient temperature evolution measured by infrared thermography.

Figure 1 illustrates the experimental setup and diagnostic methods employed in this study. As shown in Figure 1a, a CW laser (BWT, Beijing, China) with a wavelength of 976 nm and a beam diameter of 600 μm was employed as the continuous heat source. After beam collimation, the laser beam was focused onto the target surface using a focusing lens with a focal length of 100 mm, producing a laser spot with a diameter of approximately 1.2 mm. A nanosecond pulse laser (BEAMTECH, Beijing, China) with a wavelength of 1064 nm, a pulse duration of 10 ns, and a repetition rate of 5 Hz was employed as the pulse laser source. A relatively low repetition rate allows the molten pool and plasma generated by each pulse to partially decay before the arrival of the next pulse, enabling clearer identification of individual pulse-induced dynamics using high-speed imaging and infrared thermography. The laser beam was reflected by a high-reflectivity mirror and subsequently focused onto the target surface through a focusing lens with a focal length of 1000 mm. The incident angle between the CW laser beam and the ns pulse laser beam was maintained at 15°, as illustrated in Figure 1c. The incident angle was fixed at 15° to ensure stable spatial overlap between the CW and ns laser beams while minimizing reflection losses and asymmetric energy deposition. Although the incident angle can influence the effective absorption area and molten flow direction, this work mainly focuses on the synergistic interaction mechanism under a fixed beam geometry.

To ensure accurate spatial overlap of the two laser beams, the ns pulse laser was initially operated at low pulse energy to identify its focal position on the specimen surface. The reflection mirror and the three-axis translation stage were then carefully adjusted until the irradiation center of the ns pulse laser completely coincided with that of the CW laser. After the spatial overlap of the two beams had been confirmed, the pulse energy of the ns laser was increased to the preset experimental value, and the combined pulse laser irradiation experiments were subsequently carried out. The target material was a 5052-aluminum alloy plate with dimensions of 100 mm × 100 mm × 5 mm, which was mounted on a three-axis translation stage, as illustrated in Figure 1b. The 5 mm thickness is sufficient to avoid complete penetration during the investigated irradiation conditions and minimize the influence of backside heat dissipation. The large lateral dimensions provide sufficient spacing between neighboring irradiation regions, preventing thermal accumulation and interaction between adjacent experiments. Under the selected experimental conditions, the ns pulse laser generated an air plasma filament along its propagation path. Before each experiment, the position and length of the luminous filament were first identified under low-energy irradiation. The specimen was subsequently translated until the overlap region of the CW laser beam and the ns pulse laser beam coincided with the middle section of the plasma filament, where the filament propagation remained relatively stable and the laser intensity distribution was more uniform. Since the molten pool depth produced by the CW laser was approximately 0.1–0.5 mm, which was significantly smaller than the filament length, the molten surface remained within the effective focal region of the ns pulse laser throughout the irradiation process. Therefore, identical beam-overlap and filament-alignment conditions were maintained in all experiments to ensure good repeatability and consistent laser–material interaction.

To investigate the transient interaction process, an infrared thermography system (KLEIBER KMGA740-LO, Bremen, Germany) was employed to monitor the real-time surface temperature evolution of the specimen, as shown in Figure 1f. A high-speed imaging system (SSZN SH3 103, Shenzhen, China) was used to capture the dynamic evolution of plasma expansion, molten metal ejection, and material removal during laser irradiation, as presented in Figure 1e. The temporal relationship between the CW laser and the ns pulse laser was controlled using the pulse timing sequence shown in Figure 1f. After laser irradiation, the three-dimensional surface morphology, topographical profiles, and damage depth were characterized using a three-dimensional optical profilometer (KEYENCE VR-6100, Osaka, Japan). Subsequently, damage depth, material removal volume and material removal rate (MRR) were compared on the basis of the characterized three-dimensional parameters, so as to further elucidate the correlation between processing parameters and material removal performance.

In this study, MRR was defined as the removed material volume per unit input laser energy and calculated according to Equation (X) [47]:

$$MRR=\;V/E_{in}\;$$

where *MRR* represents the material removal rate (mm^3^/J), *V* is the removed volume of the laser-induced crater (mm^3^), and *Ein* is the total incident laser energy (J). For the CW–ns combined pulse laser process, the total input energy was calculated by considering the contributions from both the continuous-wave laser and nanosecond pulse laser [47]:

$$Ein\;=P_{cw}\;t+E_{ns}N$$

where *P_CW_* represents the CW laser power (W), *t* is the laser irradiation duration (s), *Ens* is the energy of a single ns pulse (J), and *N* represents the number of ns pulses during irradiation. The removed crater volume (V) was obtained from three-dimensional surface morphology measurements after laser processing. The laser-induced crater was characterized using a three-dimensional optical profilometer, and the removed volume was calculated by integrating the depth distribution of the crater relative to the original unprocessed surface.

## 3. Results and Discussions

### 3.1. Laser-Induced Damage Characteristics of 5052 Aluminum Alloy

#### 3.1.1. Comparison of Damage Morphologies Under Different Irradiation Modes

**Figure 2:**
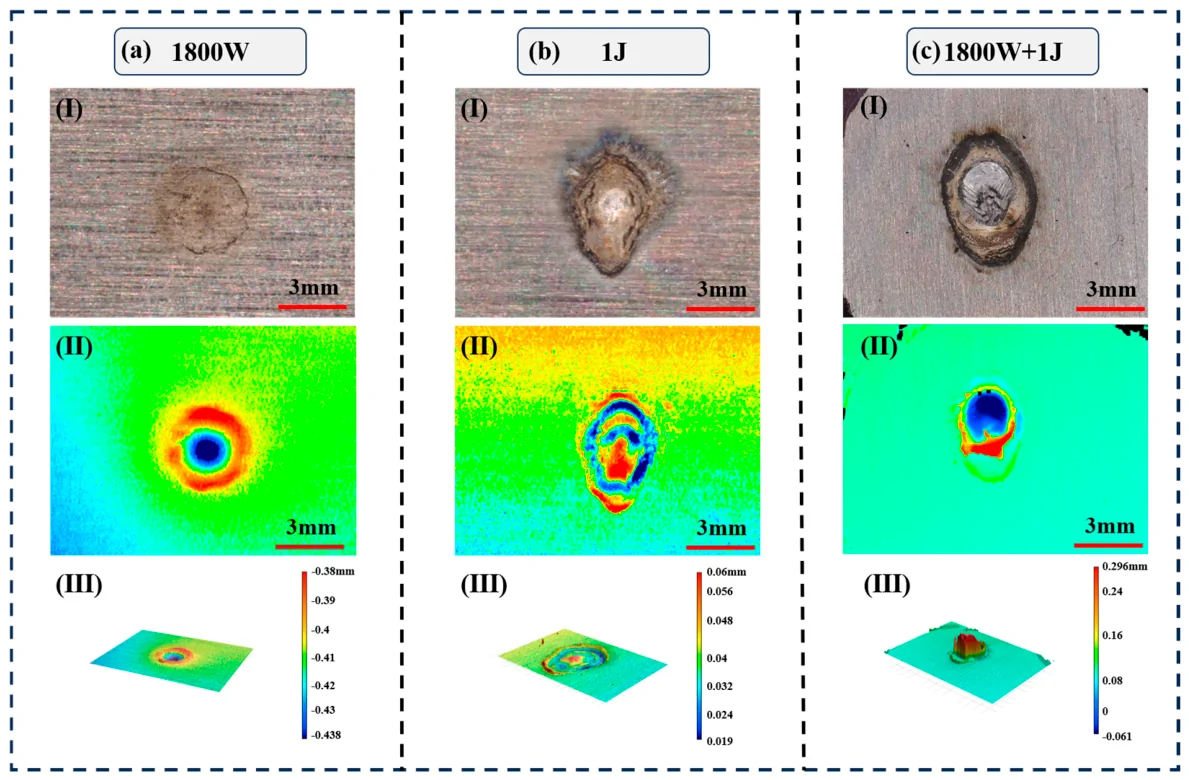
Comparison of the three-dimensional surface morphologies of 5052 aluminum alloy after irradiation by different laser modes: (**a**) CW laser irradiation (1800 W); (**b**) ns pulse laser irradiation (1 J); (**c**) combined pulse laser irradiation.

Figure 2 presents the surface morphologies of 5052 aluminum alloy irradiated under different laser conditions after 20 s exposure, including individual CW laser irradiation, ns pulse laser irradiation, and combined pulse laser irradiation. Under CW laser irradiation with a power of 1800 W, only slight surface damage was observed. Continuous thermal input from the CW laser induced a shallow and smooth molten zone with a nearly circular shape. The transition between the molten region and the unaffected area was relatively gradual, and no obvious recast layer was formed. Meanwhile, a faint blue oxidation spot appeared on the surface due to continuous thermal oxidation, as shown in Figure 2(aI–aIII). Under ns pulse laser irradiation with a pulse energy of 1 J, a droplet-like damage morphology was formed. As shown in Figure 2(bI), intense ablation occurred at the center due to the transient high-energy deposition of the ns laser. A large amount of resolidified molten material accumulated and curled around the periphery, forming a pronounced recast layer. Severe surface oxidation was also observed, resulting in a darkened appearance. The morphology was highly asymmetric, and the surface contour exhibited distorted and irregular features without a symmetrical ring structure, indicating significant lateral melt expulsion and material splashing induced by the ns laser pulse, as shown in Figure 2(bII,bIII). Under combined pulse laser irradiation (CW laser power of 1800 W and ns pulse energy of 1 J), the surface damage was further intensified. A deep crater formed at the center of the molten zone. The molten aluminum in the central region rapidly solidified into a silver-white re-solidified zone after cooling. Around the crater, a large amount of resolidified material accumulated due to the combined effects of CW thermal input and ns-induced recoil pressure, driving molten flow and outward material redistribution. Severe oxidation spots were also observed around the damaged region. The surface contour maps revealed a more regular and well-defined circular crater, surrounded by a distinct ring-shaped accumulation of molten material. The three-dimensional morphology further confirmed the pronounced build-up of the recast layer, as shown in Figure 2(cI–cIII).

#### 3.1.2. Damage Profile and Depth Evolution Under Different Irradiation Modes

**Figure 3:**
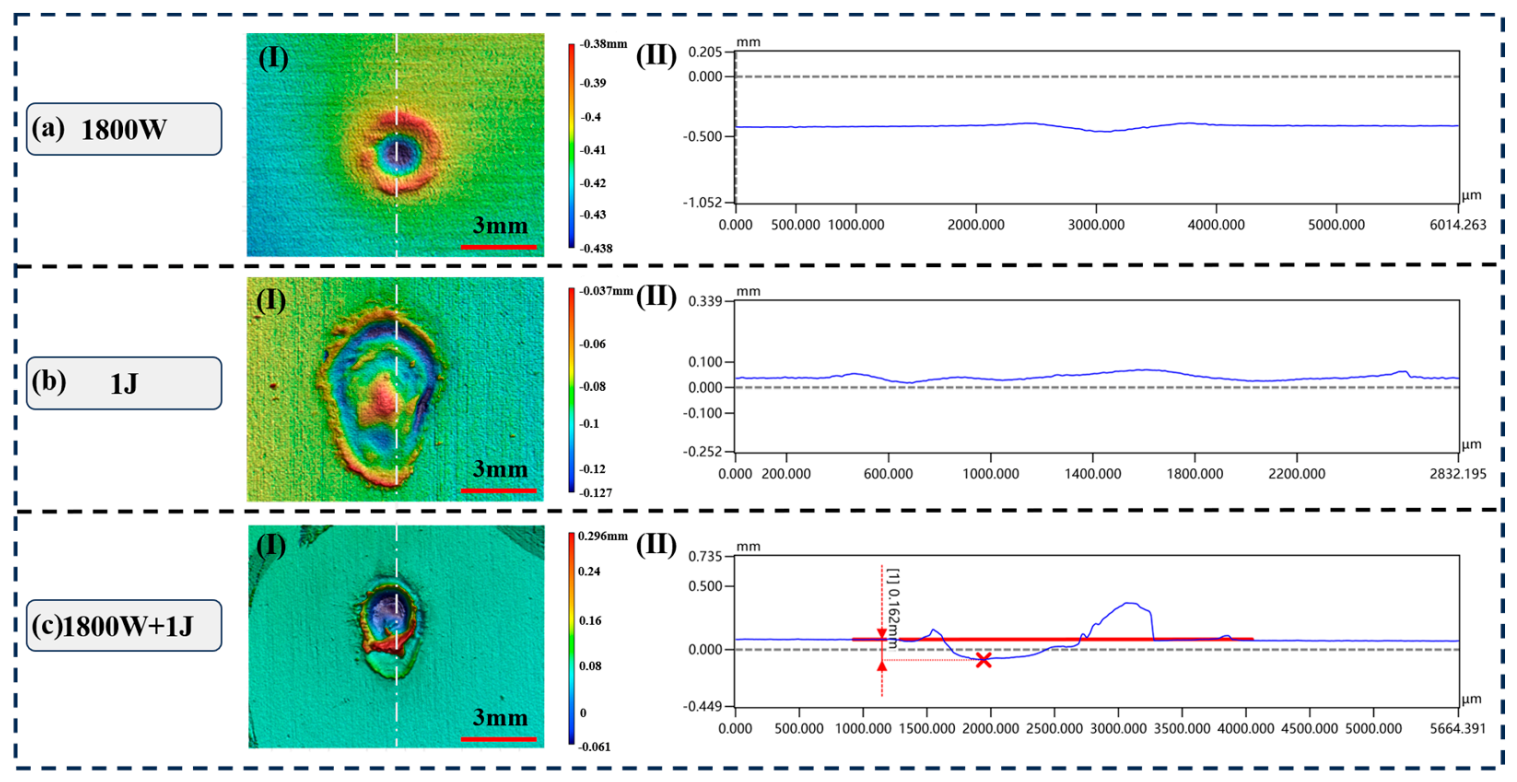
Comparison of the damage depth of 5052 aluminum alloy under CW, ns, and combined pulse laser irradiation: (**a**) CW laser irradiation (1800 W); (**b**) ns pulse laser irradiation (1 J); (**c**) combined pulse laser irradiation.

Figure 3 compares the damage morphologies and corresponding cross-sectional depth profiles along the centerline of 5052 aluminum alloy after 20 s of irradiation under individual CW laser irradiation, individual ns pulse laser irradiation, and combined pulse laser irradiation. As shown in Figure 3a, under CW laser irradiation with a power of 1800 W, the centerline profile remained nearly flat, exhibiting only a shallow depression with no obvious material accumulation or splashing. The slight uplift on both sides of the molten region and the relatively low surface roughness indicate that the continuous thermal input produced stable melting with limited material removal capability. In contrast, under ns pulse laser irradiation with a pulse energy of 1 J, the cross-sectional profile exhibited pronounced fluctuations, as shown in Figure 3b. A slight central protrusion was followed by shallow depressions and elevated recast layers on both sides, resulting in a characteristic “M”-shaped profile. Although the penetration depth remained limited, the damage width increased significantly compared with that produced by the CW laser. This morphology indicates that the transient high-peak-power pulse induced rapid melting, vaporization, and lateral ejection of molten material, accompanied by the formation of recast layers around the damaged region. Under combined pulse laser irradiation (CW laser power of 1800 W and ns pulse energy of 1 J), a markedly different damage profile was obtained, as illustrated in Figure 3c. A steep and deep central crater was formed, accompanied by pronounced accumulation of resolidified molten material around the crater edge. The maximum damage depth reached 0.162 mm, which was substantially greater than that obtained under either CW or ns laser irradiation alone. In addition, a large amount of recast material accumulated around the crater owing to the outward flow and re-solidification of molten aluminum.

A comparison of the three irradiation modes clearly demonstrates that combined pulse laser irradiation significantly enhances the material removal capability compared with either individual CW or ns pulse laser irradiation. During the combined process, the continuous thermal input from the CW laser continuously heats and melts the aluminum alloy, thereby reducing the resistance of the material to removal [48]. Subsequently, the transient high-energy ns laser pulse generates strong recoil pressure and plasma expansion, which efficiently expel the molten metal from the molten pool. The synergistic interaction between continuous thermal softening and transient pulse impact markedly improves material removal efficiency and results in deeper damage with more pronounced material ejection [49].

**Figure 4:**
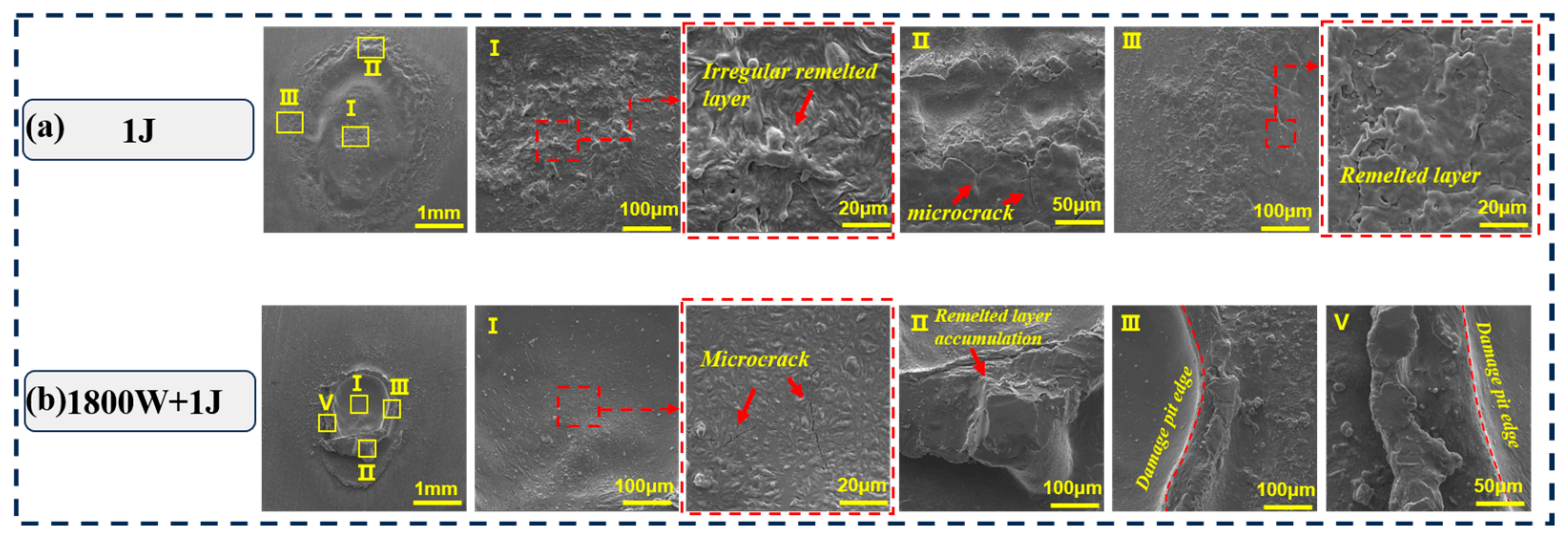
SEM characterization of surface morphology and microstructural features under different laser irradiation conditions: (**a**) ns pulse laser irradiation (1 J); (**b**) combined pulse laser irradiation (1800 W + 1 J).

The macroscopic damage morphologies and depth profiles presented above demonstrate that the irradiation mode strongly affects the material removal behavior of 5052 aluminum alloy. To further characterize the associated microstructural evolution, SEM observations were performed under representative ns pulse and CW–ns combined pulse laser conditions, as shown in Figure 4. For CW irradiation, no pronounced surface morphological variation was observed within the investigated region; therefore, its SEM image is not included. Under ns pulse irradiation at 1 J, the surface exhibits heterogeneous and irregular remelted/resolidified features accompanied by localized microcracks. These characteristics are attributed to the intense transient energy deposition, which induces rapid melting and vaporization followed by rapid cooling and solidification. The irregular remelted layer further indicates that part of the molten material is redistributed over the irradiated surface rather than being completely removed from the interaction zone.

A more pronounced microstructural evolution is observed under CW–ns combined pulse irradiation at 1800 W + 1 J. Compared with ns irradiation alone, more evident molten-material redistribution and resolidified material accumulation can be observed around the damage pit, together with localized microcracks. The coexistence of the distinct damage pit and accumulated resolidified material indicates that CW preheating modifies the initial thermal state and promotes subsequent molten-pool deformation and material ejection induced by the ns pulse. Part of the ejected molten material is subsequently redeposited and rapidly solidified around the crater. These SEM observations are consistent with the enhanced damage depth and material removal observed under combined irradiation, providing complementary microstructural evidence that CW preheating facilitates transient pulse-induced melt ejection and subsequent re-solidification.

### 3.2. Dynamic Evolution Characteristics of 5052 Aluminum Alloy Under Laser Irradiation

#### 3.2.1. CW Laser Irradiation

**Figure 5:**
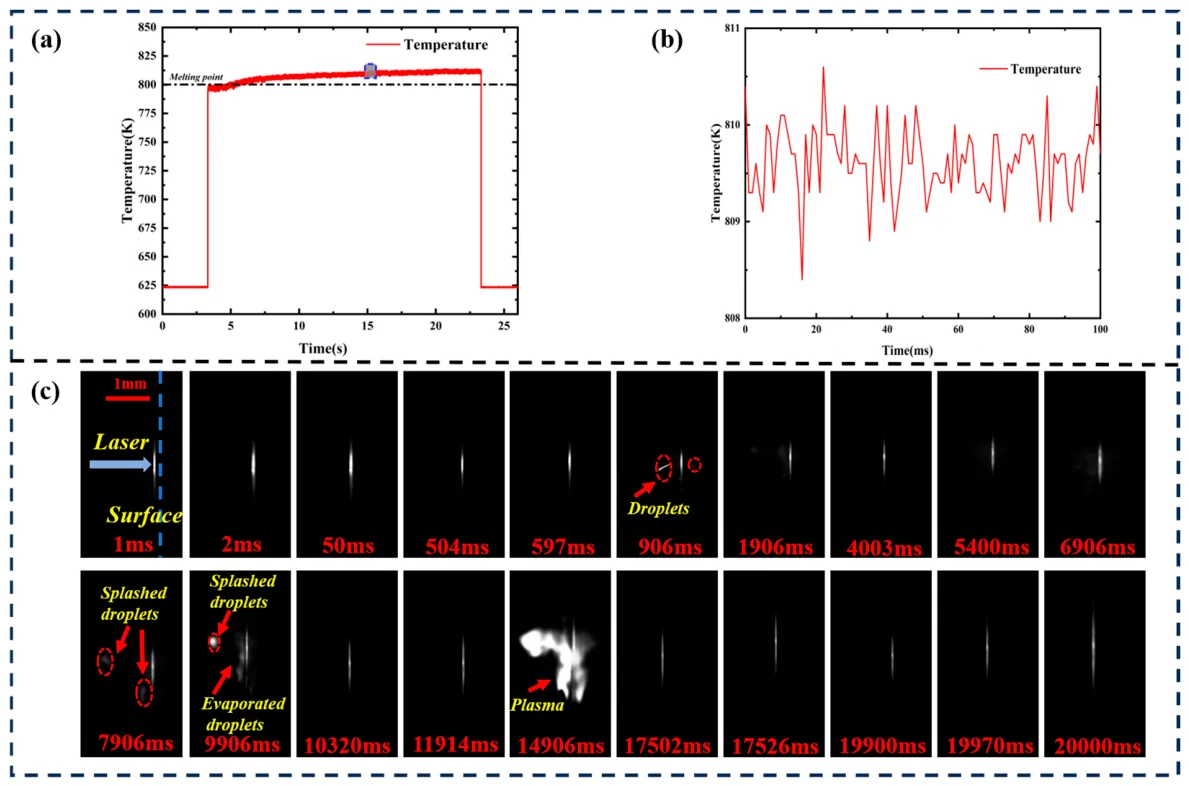
Dynamic behavior of 5052 aluminum alloy under CW laser irradiation: (**a**) transient temperature evolution; (**b**) magnified view of the selected region; (**c**) high-speed imaging results.

Figure 5 presents the transient surface temperature evolution and representative high-speed images of 5052 aluminum alloy subjected to individual CW laser irradiation at a power of 1800 W for 20 s. As shown in Figure 5a, the surface temperature increased rapidly during the initial stage of irradiation owing to continuous heat accumulation. Within the first few seconds, the temperature approached the melting temperature of 5052 aluminum alloy, leading to the formation of a stable molten pool. With continuous energy input from the CW laser, the molten pool temperature increased gradually with periodic fluctuations, while the overall temperature evolution exhibited a relatively smooth upward trend. A magnified view of the temperature curve in Figure 5b reveals repeated temperature oscillations during the irradiation process. These fluctuations can be attributed to the continuous flow of molten aluminum within the molten pool. The redistribution of molten metal intermittently exposed relatively cooler underlying material, resulting in a temporary decrease in the measured surface temperature. Continuous laser heating subsequently reheated the newly exposed surface, causing the temperature to rise again. Consequently, the temperature evolution exhibited a cyclic fluctuation superimposed on the overall increasing trend. The corresponding high-speed images shown in Figure 5c further elucidate the dynamic interaction process. Immediately after laser irradiation, a bright luminous spot appeared on the target surface, indicating rapid energy absorption and the initiation of melting. As the CW laser irradiation continued, the molten pool expanded gradually, accompanied by limited molten metal spattering and occasional vaporized droplets induced by recoil pressure. The intermittent ejection of molten material exposed fresh molten surfaces, which is consistent with the periodic temperature fluctuations observed in Figure 5b. Throughout the irradiation process, only a small amount of molten metal ejection and weak vapor emission were observed, while no obvious plasma plume or violent material expulsion occurred. These observations indicate that material removal under CW laser irradiation is dominated by a continuous thermal melting mechanism rather than by impulsive mechanical removal. Owing to the relatively low laser energy coupling efficiency and the rapid heat dissipation of aluminum alloy, the material removal capability of the CW laser remained limited. As demonstrated in Figure 2, only a shallow molten crater was formed on the specimen surface after 20 s of irradiation.

Overall, the combined analysis of high-speed imaging and real-time temperature measurements demonstrates that individual CW laser irradiation primarily induces stable thermal melting with limited material removal efficiency. Therefore, further enhancement of the damage capability requires optimization of the laser irradiation strategy, such as the introduction of transient high-peak-power laser pulses.

#### 3.2.2. Nanosecond Pulse Laser Irradiation

**Figure 6:**
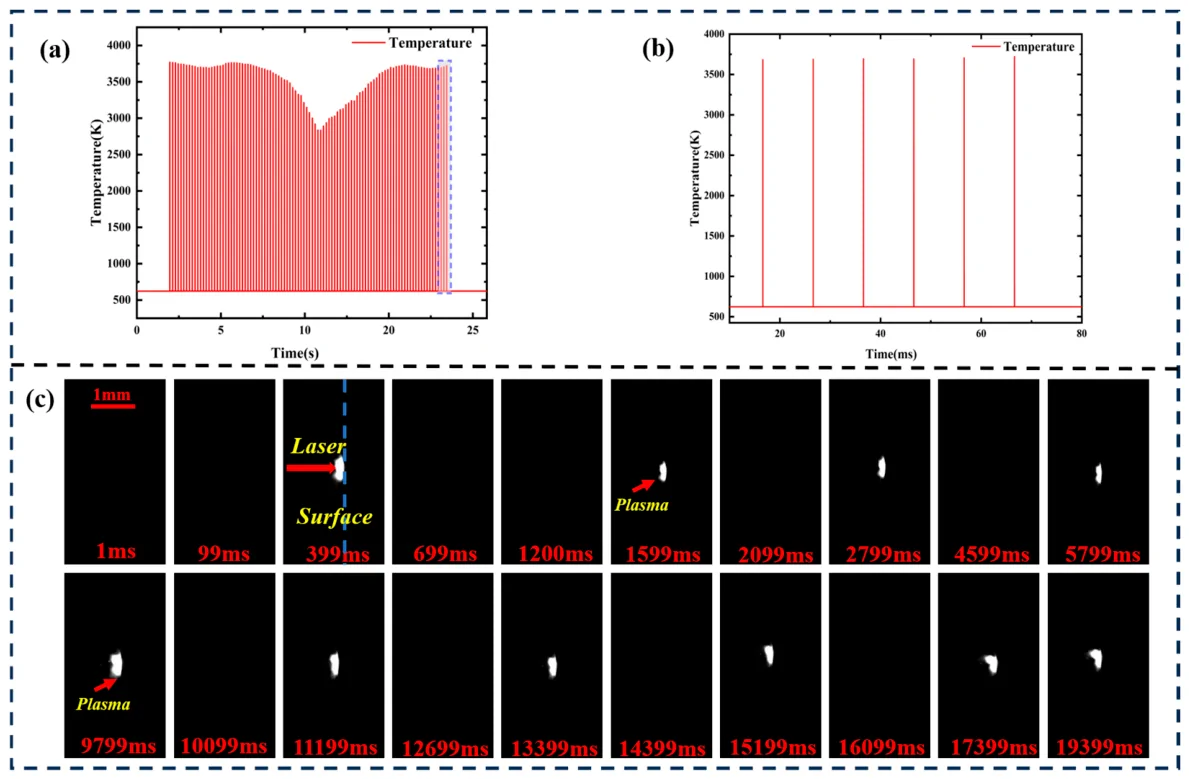
Dynamic behavior of 5052 aluminum alloy under ns pulse laser irradiation: (**a**) transient temperature evolution; (**b**) magnified region of interest; (**c**) high-speed imaging results.

Compared with individual CW laser irradiation, individual ns pulse laser irradiation exhibited distinctly different temperature evolution characteristics and dynamic interaction behavior. As shown in Figure 6a, the surface temperature displayed a typical periodic pulse-like oscillation. During each laser pulse, the surface temperature increased sharply to a peak of approximately 3700–3800 K, followed by a rapid decrease to a much lower level within a very short period. The peak temperature greatly exceeded both the melting and boiling temperatures of 5052 aluminum alloy, indicating that the material experienced not only rapid melting but also intense vaporization during each ns laser pulse, thereby providing sufficient vapor species for plasma generation. Throughout the irradiation process, the temperature profile exhibited excellent periodicity, with nearly identical peak temperatures for successive pulses, demonstrating the high stability and repeatability of the ns laser energy output. After approximately 20 s, the temperature rapidly returned to room temperature, corresponding to the termination of laser irradiation. A magnified view of the temperature profile is presented in Figure 6b, where the periodic temperature evolution can be observed more clearly. The peak temperature remained nearly constant at approximately 3500 K, while each pulse exhibited a characteristic sharp-rise and rapid-decay profile. This behavior reflects the intrinsic thermal characteristics of ns pulse laser irradiation, namely high peak power and ultrashort interaction duration. The extremely high energy density delivered within the nanosecond pulse duration caused an instantaneous temperature rise, whereas the heat dissipated rapidly after the pulse owing to thermal diffusion, resulting in a rapid decrease in surface temperature.

The corresponding high-speed images shown in Figure 6c further reveal the transient plasma evolution during ns pulse laser irradiation. During the interval of 1–99 ms, no obvious plasma emission was observed. At approximately 399 ms, a bright plasma plume appeared immediately after pulse irradiation and subsequently disappeared within a short period. As subsequent laser pulses arrived, luminous plasma plumes repeatedly formed above the target surface, exhibiting a clustered morphology. The plasma was rapidly generated after each pulse and evolved through successive cycles of generation–expansion–decay–regeneration. Both the brightness and morphology of the plasma plume fluctuated periodically, showing excellent agreement with the oscillatory temperature profile observed in Figure 6a,b.

The combined analysis of the transient temperature evolution and high-speed imaging indicates that material removal under individual ns pulse laser irradiation is dominated by a transient thermo-mechanical ablation mechanism. The ultrahigh peak power of the ns laser induces instantaneous melting and vaporization of the aluminum alloy surface, accompanied by rapid plasma formation. The recoil pressure generated by plasma expansion drives the molten metal to flow radially outward, thereby enlarging the damaged region and promoting lateral material ejection [49]. Following each pulse, the surface temperature decreases rapidly, leading to the re-solidification of the expelled molten metal and the formation of pronounced recast layers [50]. These transient processes collectively account for the characteristic damage morphology and cross-sectional profiles presented in Figure 2 and Figure 3.

#### 3.2.3. Combined Pulse Laser Irradiation

**Figure 7:**
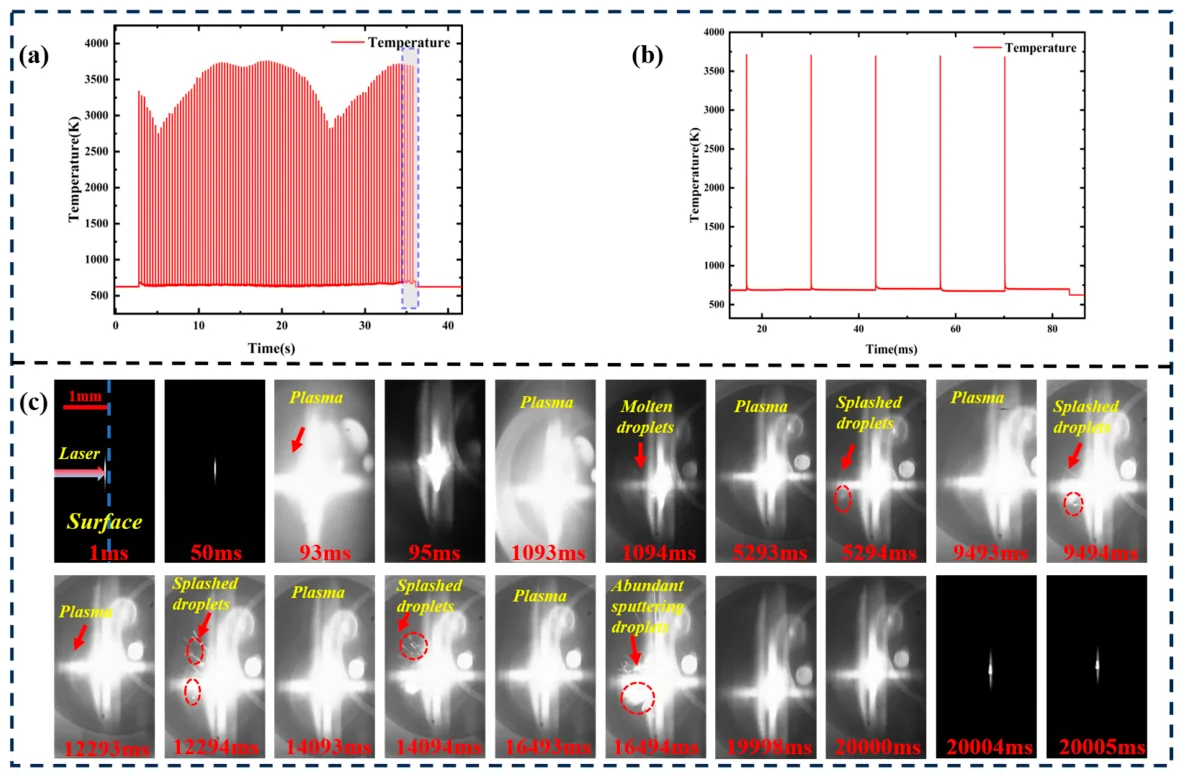
Dynamic behavior of 5052 aluminum alloy under combined pulse laser irradiation: (**a**) transient temperature evolution; (**b**) magnified view of the selected region; (**c**) representative high-speed images.

To further elucidate the transient dynamic characteristics and the underlying mechanisms responsible for the enhanced damage morphology under combined pulse laser irradiation, the same diagnostic methods, including infrared thermography and high-speed imaging, were employed, as shown in Figure 7.

The transient temperature evolution under combined pulse laser irradiation exhibited a periodic oscillatory behavior similar to that observed under individual ns pulse laser irradiation, but with several distinct characteristics. As shown in Figure 7a, during the initial stage of irradiation, the surface temperature first increased to approximately 800 K, corresponding to the preheating stage induced by the CW laser. At this stage, a stable molten pool was gradually established. Once the ns laser pulses were introduced, the surface temperature increased instantaneously to approximately 3700–3800 K during each pulse. Unlike individual ns pulse laser irradiation, where each pulse heated the material from nearly room temperature, the ns pulses in the combined pulse laser process were superimposed onto the elevated temperature field generated by continuous CW laser heating. Consequently, the molten pool remained at a relatively high temperature throughout the irradiation process, while the periodic ns pulses continuously deposited additional energy into the molten material.

A magnified view of the temperature profile is presented in Figure 7b. The pulse peak temperatures remained nearly constant, whereas the baseline temperature between adjacent pulses exhibited only slight fluctuations and remained significantly higher than that observed under individual ns pulse laser irradiation. Although each pulse still displayed the characteristic sharp-rise/rapid-decay profile, the temperature drops after each pulse became much smaller because continuous CW laser heating compensated for thermal dissipation. Owing to the high thermal conductivity of 5052 aluminum alloy, part of the absorbed thermal energy was continuously transferred into the surrounding material, thereby preventing further accumulation of the molten pool temperature despite the continuous laser energy input.

The corresponding high-speed images in Figure 7c reveal the transient evolution of plasma and molten metal ejection during combined pulse laser irradiation. At 1 ms, weak surface emission was observed immediately after laser irradiation, while a small luminous spot appeared at 50 ms, corresponding to the initial thermal radiation generated during the CW laser preheating stage. At approximately 93 ms, a bright plasma plume was formed above the molten pool. Compared with that generated by individual ns pulse laser irradiation, the plasma exhibited significantly higher brightness and a larger spatial extent, indicating that the combined laser irradiation substantially enhanced plasma generation. As the irradiation proceeded, increasingly intense molten pool dynamics were observed. At approximately 1093 ms, obvious molten droplets appeared on the molten pool surface, followed by rapid outward ejection at 1094 ms under the combined action of plasma recoil pressure and transient pulse impact, forming numerous splashed droplets. Similar periodic phenomena involving simultaneous plasma generation and molten droplet ejection were repeatedly observed at 1523 ms, 9479 ms, 12,793 ms, and 14,093 ms. By 16,493 ms, a large number of molten droplets were ejected over a much wider area, indicating extremely vigorous material removal. Plasma emission and molten metal splashing remained active until approximately 19,999–20,000 ms, whereas both phenomena disappeared rapidly after laser irradiation ceased (20,004–20,005 ms), corresponding to plasma extinction and subsequent solidification of the molten pool.

The combined analysis of the temperature evolution and high-speed imaging demonstrates that the interaction dynamics under combined pulse laser irradiation are considerably more complex than those observed under either individual CW or ns pulse laser irradiation. Continuous CW laser irradiation establishes and maintains a stable molten pool, whereas periodic ns laser pulses induce instantaneous melting, vaporization, plasma generation, and strong recoil pressure [51]. The intensified plasma expansion continuously drives molten metal outward, resulting in enhanced molten droplet ejection and more efficient material removal. This synergistic interaction between continuous thermal softening and transient pulse impact accounts for the significantly greater damage depth, larger material removal volume, and more pronounced recast morphology observed under combined pulse laser irradiation.

### 3.3. Effects of Laser Processing Parameters on the Behavior of 5052 Aluminum Alloy Under Combined Pulse Laser Irradiation

The preceding sections systematically compared the surface morphologies, transient temperature evolution, and high-speed imaging results of 5052 aluminum alloy subjected to individual CW laser irradiation, individual ns pulse laser irradiation, and combined pulse laser irradiation. The results demonstrated that combined pulse laser irradiation exhibits significantly more complex interaction dynamics and substantially higher material removal capability than either individual CW or ns pulse laser irradiation. However, the material removal performance of the combined pulse laser is strongly dependent on the processing parameters. Therefore, this section investigates the effects of different processing parameters on the damage characteristics, dynamic evolution, and material removal behavior of 5052 aluminum alloy under combined pulse laser irradiation, with the aim of clarifying the influence of CW laser power and ns pulse energy on the laser–material interaction process.

#### 3.3.1. Combined Pulse Laser Irradiation with Different CW Laser Powers

**Figure 8:**
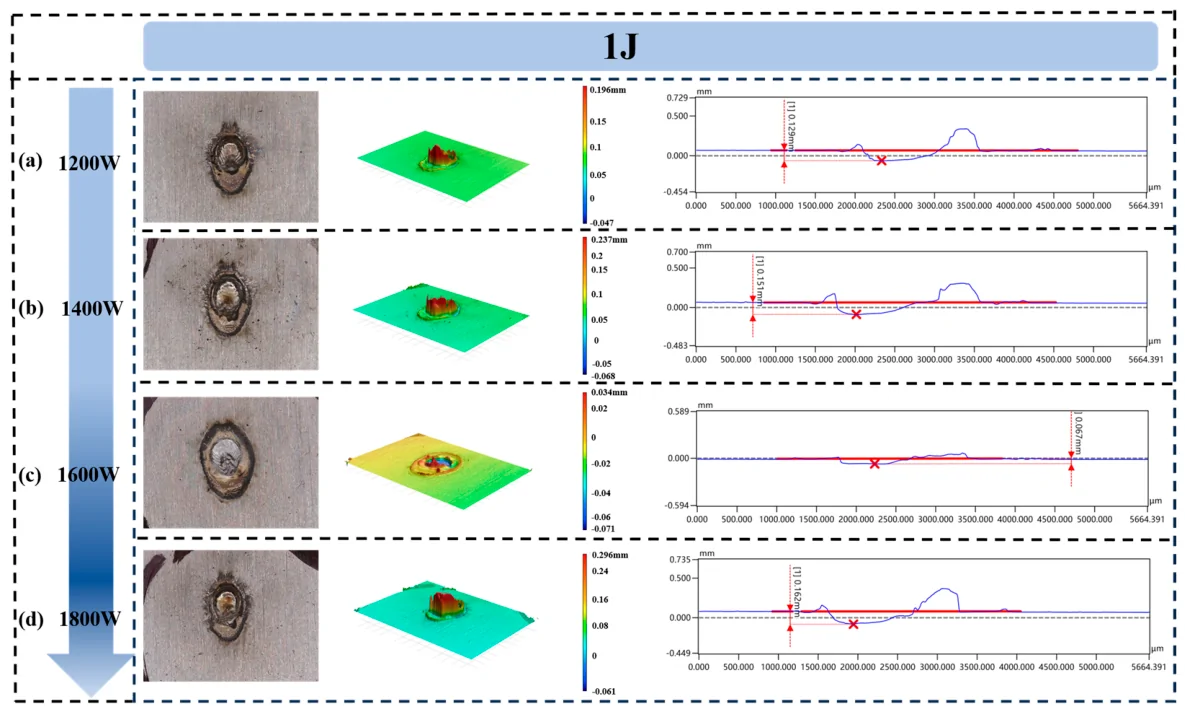
Surface morphologies of 5052 aluminum alloy under combined pulse laser irradiation with different CW laser powers at a fixed nanosecond laser energy of 1 J: (**a**) 1200 W; (**b**) 1400 W; (**c**) 1600 W; (**d**) 1800 W.

Figure 8 presents the optical morphologies, three-dimensional surface profiles, and cross-sectional depth profiles of 5052 aluminum alloy subjected to combined pulse laser irradiation with different CW laser powers at a fixed ns pulse energy of 1 J. At a CW laser power of 1200 W, a typical teardrop-shaped damage morphology was observed, as shown in Figure 8a. A central crater with a damage depth of approximately 0.129 mm was formed, surrounded by a resolidified recast layer and oxidized regions. The crater center exhibited a silver-white appearance, indicating the rapid solidification of molten aluminum after laser irradiation. As the CW laser power increased to 1400 W, the crater depth increased to 0.151 mm, accompanied by an enlarged resolidified region in the crater center. Meanwhile, the surrounding recast layer and oxidized area became more pronounced, suggesting enhanced thermal input and molten pool expansion. When the CW laser power was further increased to 1600 W, the damage depth decreased significantly to 0.047 mm, despite the observation of a larger silver-white resolidified region and a more extensive oxidized area surrounding the damaged zone. This result indicates that although the thermal influence region continued to expand, the effective material removal efficiency was substantially reduced. At the highest CW laser power of 1800 W, the damage depth increased again to 0.162 mm, which was the maximum value obtained under the investigated conditions. A deeper crater, together with a thicker recast layer and more pronounced oxidation around the crater edge, was observed, indicating a substantial enhancement in material removal capability.

Overall, the damage morphology remained similar under different CW laser powers, consisting of a central resolidified region surrounded by recast material and oxidized areas. However, the damage depth exhibited a non-monotonic variation, increasing from 1200 W to 1400 W, decreasing sharply at 1600 W, and increasing again at 1800 W. In contrast, the area of the central resolidified region first increased and then decreased, reaching a maximum when the damage depth was at its minimum. This observation suggests that the increase in CW laser power does not necessarily lead to a proportional increase in material removal efficiency.

The non-monotonic evolution of the damage depth is likely associated with the competition between thermal energy deposition and plasma-assisted energy loss. At relatively low CW laser powers, increasing thermal input promotes molten pool growth and enhances material removal. However, at intermediate power levels, intensified plasma shielding and increased energy dissipation may reduce the effective laser energy coupled into the target, resulting in a decrease in penetration depth. As the CW laser power is further increased, the interaction is likely to transition from a conduction-dominated melting regime to a deeper penetration mode with stronger synergistic interaction between the CW laser and ns laser pulses, thereby restoring and further enhancing the material removal capability.

#### 3.3.2. Combined Pulse Laser Irradiation with Different Nanosecond Laser Energies

**Figure 9:**
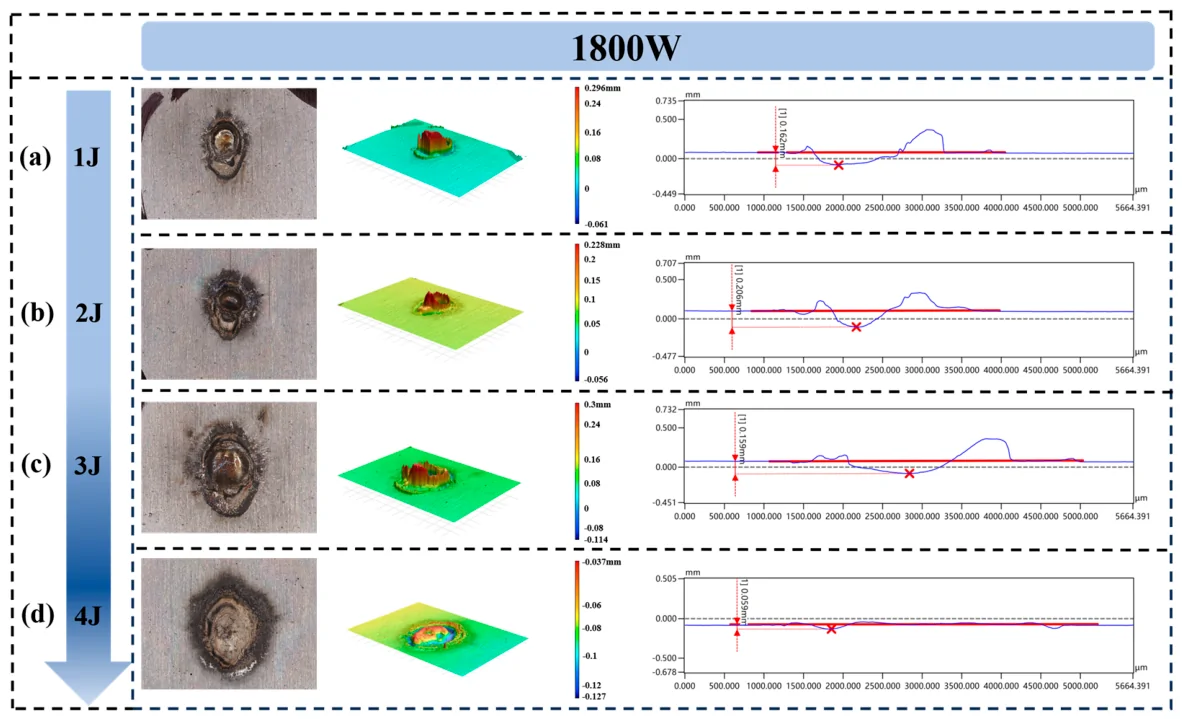
Surface morphologies of 5052 aluminum alloy under combined pulse laser irradiation with different nanosecond laser energies at a fixed CW laser power of 1800 W: (**a**) 1 J; (**b**) 2 J; (**c**) 3 J; (**d**) 4 J.

Figure 9 presents the optical morphologies, three-dimensional surface profiles, and cross-sectional depth profiles of 5052 aluminum alloy subjected to combined pulse laser irradiation with different ns pulse energies at a fixed CW laser power of 1800 W. At a pulse energy of 1 J, a relatively small molten spot with a shallow central crater was formed, corresponding to a damage depth of approximately 0.162 mm, while the surrounding oxidized region remained limited, as shown in Figure 9a. When the pulse energy was increased to 2 J, the diameter of the molten region increased noticeably, and the central crater deepened to 0.206 mm, representing the maximum penetration depth obtained under the investigated conditions. The crater exhibited a more regular circular geometry, accompanied by a well-defined recast ring around its periphery, indicating the highest material removal efficiency and the most stable damage morphology. With a further increase in pulse energy to 3 J, the molten region continued to expand, whereas the regularity of the central crater gradually deteriorated. More resolidified material and splashing traces accumulated around the crater, while the damage depth decreased to approximately 0.159 mm, suggesting that the increase in pulse energy no longer resulted in improved penetration. At the highest pulse energy of 4 J, the molten spot reached its maximum lateral dimension, and the oxidized region became considerably wider. However, the central crater became significantly shallower, with the damage depth decreasing sharply to only 0.059 mm. In addition, severe molten metal splashing and extensive recast layer accumulation were observed, indicating substantial deterioration of the damage morphology despite the larger thermally affected area.

Overall, the lateral size of the damaged region increased continuously with increasing ns pulse energy, whereas the penetration depth exhibited a non-monotonic trend, increasing initially and then decreasing at higher pulse energies. This behavior suggests that increasing pulse energy does not necessarily improve the material removal efficiency under combined pulse laser irradiation [52,53]. The reduction in penetration depth at high pulse energies is likely associated with intensified plasma shielding and increasingly violent molten pool dynamics. As the pulse energy increases, stronger plasma generation can absorb and scatter a larger fraction of the incident laser energy, thereby reducing the effective energy delivered to the material surface. Meanwhile, the enhanced recoil pressure induces vigorous molten pool oscillation and molten metal ejection. Part of the expelled molten material subsequently resolidifies and re-deposits inside or around the crater, partially filling the damaged region. Furthermore, an increasing proportion of the laser energy is consumed by excessive vaporization and plasma expansion rather than effective material removal [54], ultimately leading to a reduction in penetration depth despite the higher pulse energy.

#### 3.3.3. Dynamic Evolution Under Combined Pulse Laser Irradiation with Different CW Laser Powers

**Figure 10:**
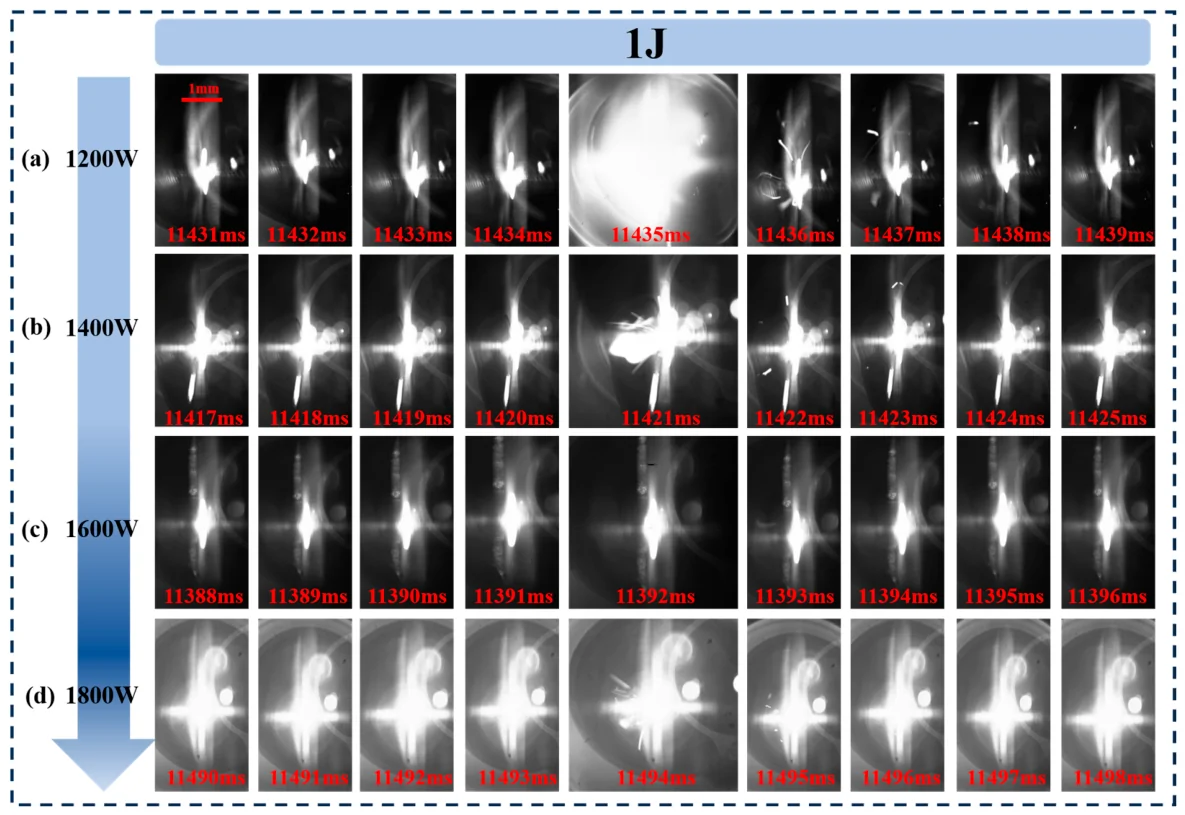
High-speed images of 5052 aluminum alloy at t = 11 s under combined pulse laser irradiation with different CW laser powers and a fixed nanosecond laser energy of 1 J: (**a**) 1200 W; (**b**) 1400 W; (**c**) 1600 W; and (**d**) 1800 W.

To further elucidate the mechanism by which the CW laser power influences the damage depth, Figure 10 compares the high-speed images of 5052 aluminum alloy irradiated by the combined pulse laser at a fixed ns pulse energy of 1 J with CW laser powers ranging from 1200 to 1800 W. The images were recorded at 11 s, when the laser–material interaction had reached a relatively stable state. As the CW laser power increased from 1200 W to 1800 W, the brightness and spatial extent of the plasma plume exhibited a distinct enhancement–attenuation–re-enhancement trend, which closely corresponded to the variation in damage depth observed in Figure 8. At 1200 W and 1400 W, bright plasma plumes were generated immediately after each ns laser pulse and were accompanied by obvious molten droplet ejection. Compared with 1200 W, the plasma emission at 1400 W became noticeably brighter, while the number of splashed molten droplets also increased, indicating enhanced laser–material interaction and more efficient material removal. When the CW laser power was further increased to 1600 W, the plasma emission weakened considerably and was replaced by a relatively stable luminous molten pool. Meanwhile, both plasma expansion and molten droplet ejection became much less pronounced than those observed at lower laser powers. Correspondingly, the damage depth decreased significantly, suggesting a reduction in the effective material removal efficiency. At 1800 W, the plasma plume became significantly brighter again and exhibited the largest spatial extent among all investigated conditions. Simultaneously, violent molten metal ejection and the formation of numerous large splashed droplets were observed, indicating that the interaction between the CW laser and the ns laser pulses was substantially intensified. This enhanced interaction resulted in the greatest material removal capability and the maximum damage depth.

By comparing the high-speed images with the corresponding damage morphologies, it can be inferred that the evolution of the plasma plume and molten pool dynamics plays a dominant role in determining the material removal behavior under combined pulse laser irradiation. At relatively low CW laser powers (1200–1400 W), increasing thermal input promotes molten pool formation and metal vaporization, leading to stronger plasma generation and enhanced recoil pressure. The combined action of plasma expansion and ns pulse impact facilitates molten metal ejection, thereby increasing the damage depth. However, at 1600 W, the interaction behavior changes markedly. Although the molten pool becomes larger because of the increased thermal input, the plasma emission is significantly weakened and molten metal ejection is suppressed. These observations suggest that the effective coupling between the ns laser pulses and the molten pool is reduced, resulting in less efficient material removal despite the larger molten region. When the CW laser power is further increased to 1800 W, both plasma generation and molten metal ejection recover and become more intense. The stronger plasma recoil pressure promotes vigorous molten metal expulsion from the crater, thereby restoring the material removal efficiency and producing the deepest damage. Therefore, the increase–decrease–increase trend of the damage depth with increasing CW laser power can be attributed to changes in the laser energy coupling behavior, which regulate plasma evolution, molten pool dynamics, and molten metal ejection during the combined pulse laser process.

#### 3.3.4. Dynamic Evolution Under Combined Pulse Laser Irradiation with Different Nanosecond Laser Energies

**Figure 11:**
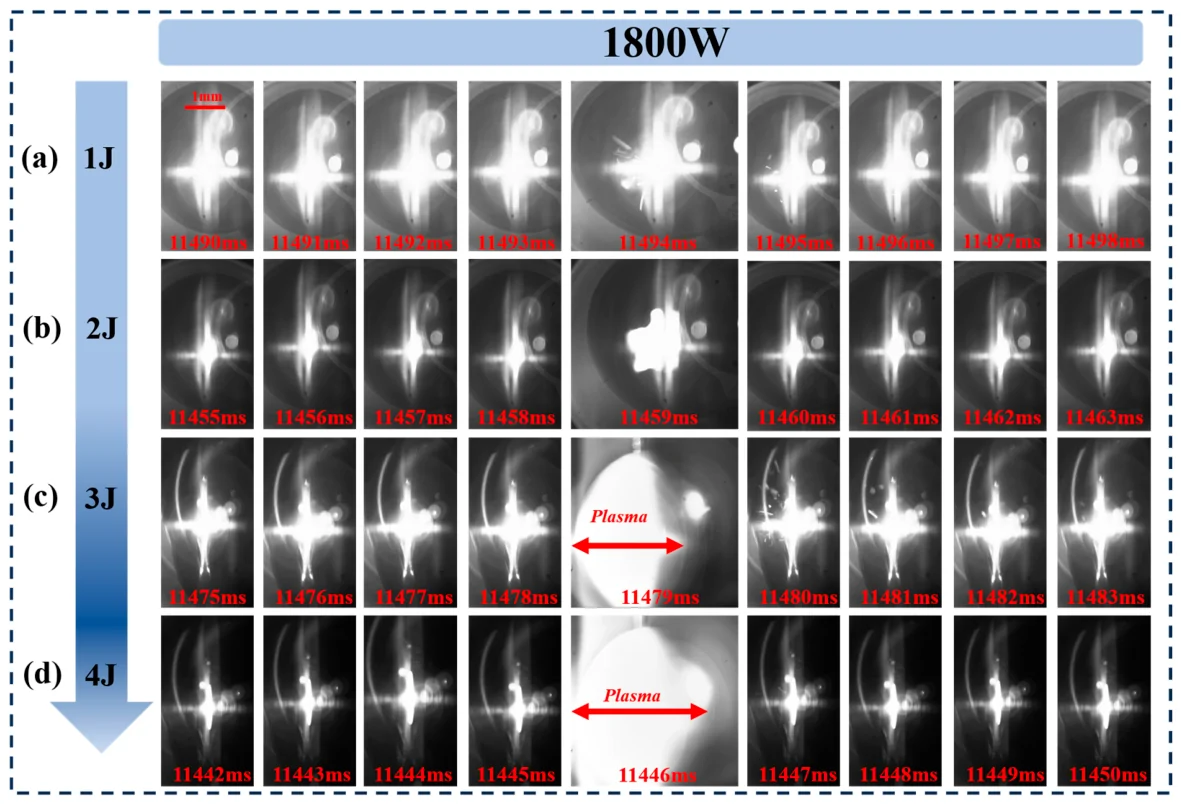
High-speed images of 5052 aluminum alloy at t = 11 s under combined pulse laser irradiation with different nanosecond laser energies and a fixed CW laser power of 1800 W: (**a**) 1 J; (**b**) 2 J; (**c**) 3 J; (**d**) 4 J.

To further clarify the influence of ns pulse energy on the laser–material interaction process, Figure 11 compares the high-speed images of 5052 aluminum alloy irradiated by the combined pulse laser with ns pulse energies ranging from 1 to 4 J at a fixed CW laser power of 1800 W. The images were recorded at 11 s, corresponding to the stable stage of the interaction process.

Under all investigated conditions, bright plasma plumes accompanied by molten metal ejection were observed. At relatively low pulse energies (1–2 J), the continuous thermal input from the CW laser maintained a stable molten pool, while each ns pulse generated a bright plasma plume and promoted the radial ejection of molten droplets from the interaction zone. Compared with 1 J, the plasma brightness and the number of splashed droplets increased at 2 J, indicating enhanced laser–material coupling and more efficient molten metal removal. When the pulse energy increased to 3 J, the plasma expanded considerably, forming a larger and brighter plasma cloud above the molten pool. Simultaneously, the lateral extent of the plasma increased noticeably, accompanied by a larger number of molten droplets distributed around the interaction zone. At the highest pulse energy of 4 J, the plasma reached its maximum intensity and occupied almost the entire field of view. The interaction region was nearly completely covered by a dense luminous plasma cloud, while molten metal splashing became increasingly violent. Compared with the lower pulse energies, the plasma morphology evolved from a relatively jet-like structure to a cluster-like expanding cloud, indicating a substantial change in the laser–plasma interaction behavior.

Interestingly, this evolution of the plasma morphology closely corresponds to the variation in damage depth shown in Figure 9, where the penetration depth first increases and subsequently decreases with increasing pulse energy. At relatively low pulse energies (1–2 J), the generated plasma appears to enhance the interaction process. The plasma expansion may provide additional thermal feedback to the molten pool, while the recoil pressure generated by plasma expansion, together with the transient impact of the ns laser pulse, facilitates molten metal ejection. Under these conditions, plasma shielding remains relatively weak, allowing most of the laser energy to be effectively coupled into the molten pool. As the pulse energy increases beyond 2 J, the plasma expands rapidly and forms a thicker plasma cloud above the molten pool. The enlarged plasma is expected to absorb, scatter, and partially deflect a greater fraction of the incident laser energy, thereby reducing the effective energy delivered to the material surface. Meanwhile, a larger proportion of the deposited energy is consumed by plasma expansion and excessive vaporization rather than effective material removal. Consequently, although the molten pool continues to expand laterally, the penetration depth gradually decreases. At 4 J, the dense plasma cloud becomes even more pronounced, suggesting that plasma shielding plays a dominant role in the interaction process. Under this condition, the effective laser energy reaching the molten pool is further reduced despite the higher incident pulse energy. In addition, the intensified recoil pressure induces vigorous molten pool oscillation and severe molten metal splashing, resulting in substantial recast layer accumulation and deterioration of the damage morphology. Therefore, the material removal capability decreases significantly even though the plasma intensity reaches its maximum.

Overall, these results demonstrate that increasing the ns pulse energy does not continuously improve the material removal performance during combined pulse laser irradiation. Instead, an optimum pulse energy exists. At relatively low pulse energies, insufficient transient impact limits the material removal efficiency, whereas excessive pulse energy promotes strong plasma shielding and unstable molten pool dynamics, thereby reducing the effective laser energy utilization and ultimately decreasing the penetration depth. Consequently, controlling the plasma in a weak-shielding but strong-assistance regime is essential for achieving efficient material removal and high-quality laser processing.

These observations indicate that the role of plasma during combined pulse laser irradiation is dual in nature. Moderate plasma generation enhances material removal through thermal assistance and recoil pressure, whereas excessive plasma evolution suppresses laser energy coupling via plasma shielding [55]. The competition between these two effects ultimately determines the material removal efficiency under different ns pulse energies.

### 3.4. Comparison of Laser-Induced Damage Performance Under Different Processing Parameters

**Figure 12:**
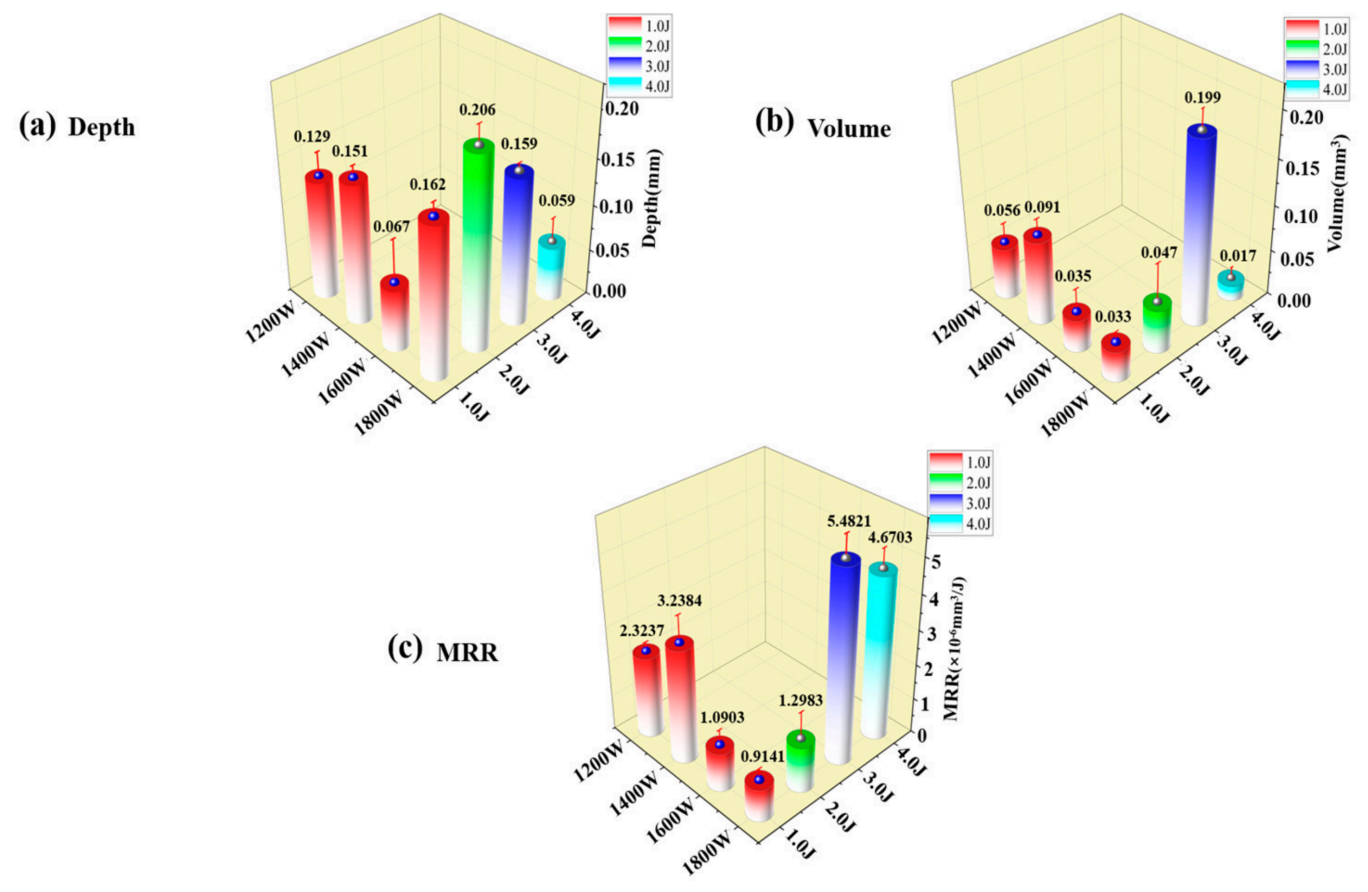
Comparison of laser-induced damage performance under different processing parameters: (**a**) damage depth; (**b**) removed volume; (**c**) material removal rate (MRR).

Figure 12 summarizes the damage performance of 5052 aluminum alloy under different combined pulse laser processing parameters by comparing the damage depth, material removal volume, and material removal rate (MRR). Combined with the morphology analysis and high-speed imaging presented in Section 3.3, the relationships between processing parameters and material removal performance can be further clarified.

Under a fixed ns pulse energy of 1 J, increasing the CW laser power from 1200 W to 1800 W resulted in a non-monotonic variation in the damage depth. Specifically, the penetration depth increased from 0.129 mm at 1200 W to 0.151 mm at 1400 W, decreased markedly to 0.067 mm at 1600 W, and subsequently increased to 0.162 mm at 1800 W, exhibiting a characteristic increase–decrease–increase trend. This behavior is consistent with the plasma evolution observed in Figure 10. At relatively low CW laser powers, increased thermal input promotes molten pool formation and plasma generation, thereby enhancing material removal. At intermediate power, the enlarged molten pool and reduced effective laser–material coupling suppress plasma-assisted material ejection, resulting in a decrease in penetration depth. With a further increase in CW laser power, stronger plasma generation and more vigorous molten metal ejection are re-established, leading to enhanced synergistic interaction and deeper penetration.

In contrast, when the CW laser power was fixed at 1800 W, increasing the ns pulse energy from 1 J to 4 J produced a single-peak evolution in the penetration depth. The maximum depth was obtained at 2 J, followed by a gradual decrease at higher pulse energies. As demonstrated by the high-speed images in Figure 11, this behavior is closely associated with the transition from plasma-assisted energy coupling to plasma shielding. Moderate plasma generation enhances molten metal ejection through recoil pressure, whereas excessive plasma expansion absorbs and scatters an increasing fraction of the incident laser energy, thereby reducing the effective energy delivered to the molten pool and suppressing material removal. The variation in the material removal volume generally follows the same trend as the penetration depth, indicating that the penetration depth is the dominant factor governing the removed volume. However, the two parameters are not completely consistent because the removal volume is also influenced by the lateral expansion of the molten pool. For example, under the condition of 1800 W–4 J, although the penetration depth reached its minimum, the removal volume did not decrease proportionally owing to the significant increase in the crater diameter.

The MRR, which characterizes the processing efficiency, exhibits trends generally similar to those of the penetration depth. Under a fixed ns pulse energy of 1 J, the MRR first increased with increasing CW laser power and then decreased at higher powers. Although the penetration depth recovered at 1800 W, the MRR remained lower than that at 1400 W, indicating that the processing efficiency is jointly affected by both the penetration depth and the crater geometry. Under a fixed CW laser power of 1800 W, the MRR also exhibited a rise-and-fall trend with increasing ns pulse energy, reaching its maximum of 5.4821 × 10^−6^ mm^3^/J at 3 J. Interestingly, although the penetration depth decreased markedly at 4 J, the MRR remained relatively high. This discrepancy suggests that, at high pulse energies, the processing mode gradually changes from a deep-and-narrow removal mode to a shallow-and-wide removal mode. The enhanced lateral expansion of the molten pool partially compensates for the reduction in penetration depth, thereby maintaining a relatively high material removal efficiency.

Overall, the results demonstrate that the CW laser power and ns pulse energy regulate the material removal performance through different physical mechanisms. The CW laser power primarily influences the molten pool evolution and laser energy coupling, resulting in a characteristic increase–decrease–increase variation in the penetration depth. In contrast, the ns pulse energy governs the competition between plasma-assisted material removal and plasma shielding, leading to a single-peak dependence of the processing performance. When the pulse energy exceeds the optimum value, plasma shielding becomes increasingly dominant, causing the penetration depth to decrease rapidly, whereas the lateral expansion of the molten pool continues to increase. Consequently, the removal volume and MRR decrease more slowly than the penetration depth, producing an apparent decoupling between different processing performance indicators. Therefore, optimizing the balance between plasma enhancement and plasma shielding by appropriately selecting the processing parameters is essential for achieving both high material removal efficiency and superior surface quality.

These results indicate that the processing performance of the combined pulse laser is governed by the synergistic evolution of the molten pool, plasma dynamics, and molten metal ejection. The competition between plasma-assisted energy deposition and plasma shielding ultimately determines the damage morphology, penetration depth, and material removal efficiency under different processing parameters.

Previous studies on laser processing of aluminum alloys have mainly focused on individual CW or ns pulse laser irradiation. CW lasers can establish a stable molten pool through continuous thermal accumulation, but their material removal capability is generally limited by relatively low peak power density and significant heat diffusion. In contrast, ns pulse lasers provide high peak power density and induce rapid melting, vaporization, and plasma generation [56]; however, their removal efficiency can be restricted by plasma shielding, molten material redistribution, and energy attenuation during laser–plasma interaction [57]. In the present study, the damage behavior of 5052 aluminum alloy under CW, ns pulse, and CW–ns combined pulse laser irradiation was systematically compared. Under identical experimental conditions, the combined pulse laser achieved a maximum damage depth of 0.206 mm, which was significantly higher than those obtained by individual CW or ns irradiation. Although direct quantitative comparison with previous studies is difficult due to differences in material properties, laser parameters, and evaluation methods, the present results demonstrate improved material removal capability compared with conventional single-laser processing strategies.

### 3.5. Process Dynamics of 5052 Aluminum Alloy Under Combined Pulse Laser Irradiation

**Figure 13:**
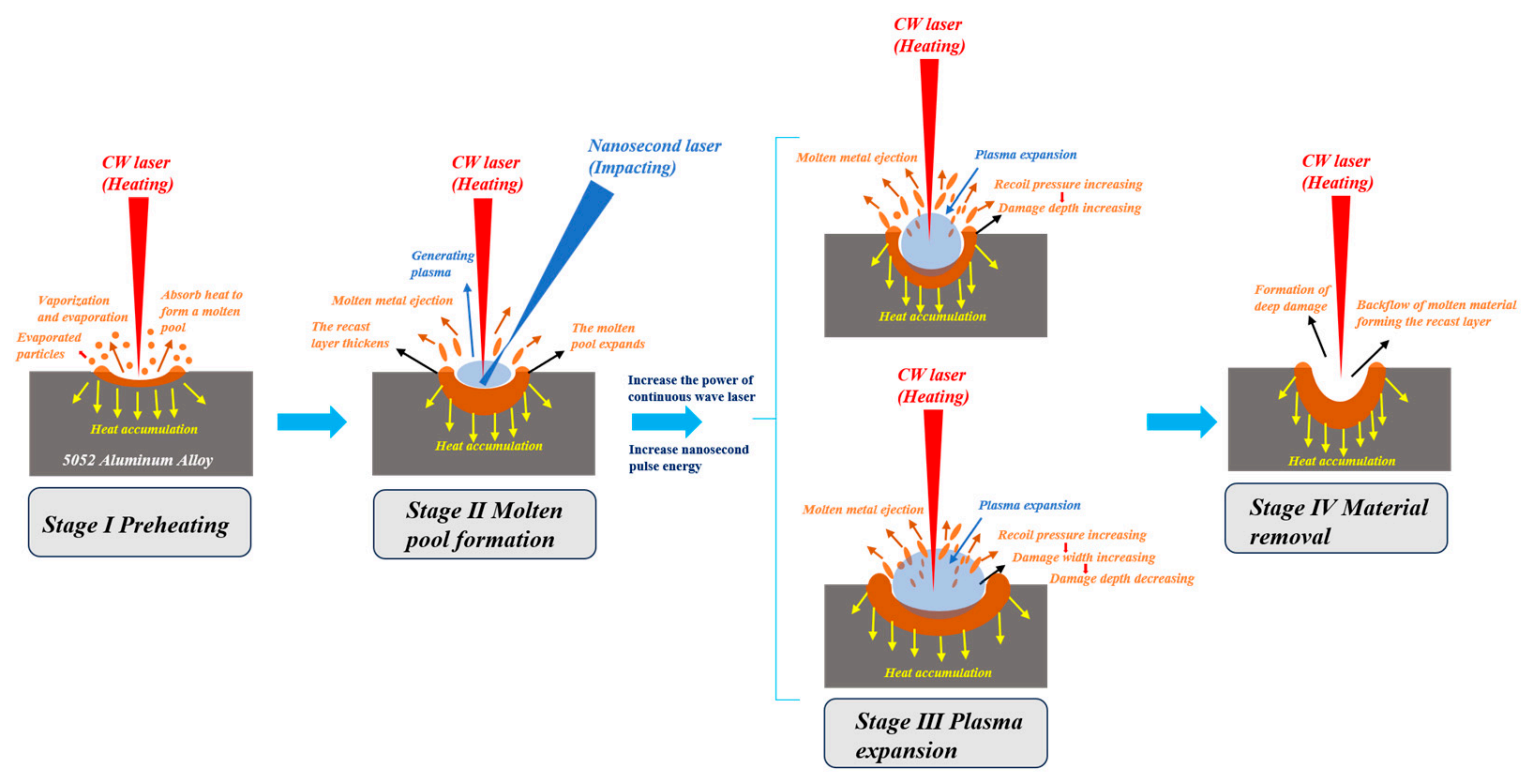
Four dynamic stages of 5052 aluminum alloy under combined pulse laser irradiation.

Figure 13 illustrates the dynamic material removal process of 5052 aluminum alloy under combined pulse laser irradiation. Based on the transient temperature evolution, high-speed imaging observations, and damage morphology analyses, the laser–material interaction can be divided into four successive stages. At the initial stage of irradiation, the CW laser first impinges on the material surface. The absorbed laser energy is converted into thermal energy and continuously conducted into the substrate. As heat accumulates, the surface temperature gradually increases from room temperature toward the melting point of the alloy [58,59]. Slight metal evaporation begins when the temperature approaches the melting point, generating a small amount of metal vapor while no significant plasma emission or material ejection is observed. During this stage, the CW laser primarily establishes a stable thermal field for the subsequent interaction with the nanosecond laser pulses. With continuous CW laser irradiation, the surface temperature exceeds the melting point, resulting in the formation of a stable molten pool. Continuous thermal input causes the molten pool to expand laterally and penetrate deeper into the substrate [53,58]. As the surface temperature further approaches the boiling point, metal vaporization intensifies. Meanwhile, the incident nanosecond laser pulses initiate plasma generation above the molten surface [59,60]. Driven by the transient impact of the nanosecond pulses and the initial plasma recoil pressure, part of the molten metal is expelled from the center of the molten pool toward the surrounding region. The ejected droplets subsequently solidify around the crater edge, forming an initial recast layer that gradually thickens with continued irradiation [61]. Following molten pool formation, the interaction enters the plasma-dominated stage. Owing to the extremely high peak power density of the nanosecond laser pulses, the molten surface is instantaneously heated to a much higher temperature within several nanoseconds, leading to rapid vaporization and explosive plasma expansion [59,60]. The high-temperature and high-pressure plasma generates a strong recoil pressure, which acts together with the transient shock induced by the laser pulse to accelerate molten metal ejection. Consequently, the molten pool expands rapidly, and the penetration depth increases significantly. The plasma behavior during this stage strongly depends on the processing parameters. Increasing the CW laser power enlarges the molten pool and modifies the laser energy distribution, thereby affecting the plasma evolution and the efficiency of molten metal ejection. Likewise, increasing the nanosecond pulse energy initially enhances plasma-assisted material removal by increasing the recoil pressure and secondary thermal input. However, when the pulse energy exceeds a critical threshold, excessive plasma expansion leads to pronounced plasma shielding, which absorbs and scatters the incident laser energy, reduces the effective energy coupled into the material, and consequently suppresses the penetration depth despite the higher laser energy input [55,59]. After repeated pulse irradiation, the molten pool gradually reaches a dynamic equilibrium between melting, ejection, and re-solidification. The crater geometry becomes relatively stable, while molten metal continuously flows along the crater wall under the combined effects of plasma recoil pressure, gravity, surface tension, and molten pool convection. The expelled molten material finally solidifies around the crater edge, forming a continuous recast layer with a characteristic morphology. At this stage, the final crater geometry and material removal characteristics are established [58,62].

Overall, the interaction between the combined pulse laser and 5052 aluminum alloy is essentially a dynamic evolution process involving progressively enhanced energy coupling and increasingly complex multi-physics interactions. The dominant mechanism evolves from pure heat conduction during the preheating stage, to coupled melting and vaporization during molten pool formation, and finally to the synergistic interaction of thermal accumulation, plasma expansion, recoil pressure, and molten metal dynamics during the plasma-dominated stage. In this process, the CW laser continuously provides thermal accumulation and maintains a stable molten pool, whereas the nanosecond laser pulses supply transient high-energy excitation that promotes plasma generation and molten metal ejection. The balance between plasma-assisted material removal and plasma shielding ultimately determines the laser energy coupling efficiency, crater morphology, penetration depth, and overall material removal performance, thereby constituting the fundamental mechanism responsible for efficient and high-quality processing under combined pulse laser irradiation. However, for metallic materials such as aluminum alloys, polarization-dependent absorption may exist, especially under conditions involving periodic surface structures or highly localized ablation [63]. However, for the relatively large laser spot size used in the present study and the randomly evolving molten pool surface, the effect of polarization is expected to be secondary compared with the dominant factors including thermal accumulation, plasma shielding, and recoil-pressure-driven molten metal ejection.

In the present study, the CW laser provides a stable thermal accumulation environment, maintaining the aluminum alloy surface near the melting state. Subsequently, nanosecond pulses interact with the preheated molten region, resulting in rapid temperature elevation, intense evaporation, plasma formation, and recoil-pressure-driven melt ejection [58]. This sequential interaction explains the enhanced damage response observed under combined irradiation compared with individual laser irradiation. Nevertheless, the enhancement effect is not unlimited. With increasing laser energy, the intensified plasma plume may act as an energy barrier, weakening the subsequent laser energy deposition into the target. Meanwhile, excessive melt ejection and unstable hydrodynamic behavior may reduce the effective material removal efficiency. Therefore, the damage evolution under CW–ns combined irradiation is determined by the balance between thermal accumulation, transient energy deposition, plasma shielding, and melt dynamics [59].

### 3.6. Comparison with Previous Studies, Limitations, and Future Perspectives

To further assess the present CW–ns combined pulse laser process, representative previous studies are summarized in Table 1. Jia et al. [63] investigated CW–ns combined laser drilling and demonstrated that the matching between CW power and ns pulse input strongly affects material removal and recast-layer formation. Compared with their work, the present study uses a similar CW–ns temporal coupling strategy but focuses on 5052 aluminum alloy and systematically evaluates the effects of CW power and ns pulse energy on damage depth, temperature evolution, and surface morphology. Thus, the present work extends the CW–ns processing concept from drilling efficiency to the detailed damage evolution of aluminum alloy. Ding et al. [52] reported that CW irradiation can modify the initial thermal state of the target and thereby enhance subsequent pulsed-laser ablation. The present results show a similar thermal-preconditioning effect, as the CW laser maintains the aluminum alloy near its melting state before ns pulse irradiation. Compared with Ding et al., the present study further demonstrates this effect through the combined analysis of temperature evolution, plasma behavior, damage morphology, and penetration depth, showing that the enhanced removal originates from the coupling between CW-induced thermal accumulation and localized ns pulse excitation rather than simply from a higher peak temperature. Yuan et al. [53] investigated 7A04 aluminum alloy using a millisecond–nanosecond combined pulse laser and reported damage depths of 125.37–149.24 μm for different spot-size configurations. In comparison, the present study employs a 1.2 mm CW spot and a 0.5 mm ns spot, and achieves a maximum damage depth of 206 μm, approximately 38.0% higher than the maximum value reported by Yuan et al. Although the two studies differ in alloy composition and laser parameters, the comparison indicates an improved penetration capability under the present experimental conditions. More importantly, it highlights the potential advantage of combining a relatively broad CW thermal field with a localized ns energy input for regulating laser-induced material removal.

More recently, Guo et al. [34] and Li et al. [47] extended combined-laser processing toward CW–femtosecond filament-assisted irradiation of ceramic materials. Guo et al. reported a damage efficiency of approximately 1.51 × 10^7^ μm^3^/J using 500 W CW irradiation combined with a 1 mJ femtosecond filament, while Li et al. further demonstrated an ablation efficiency on the order of 10^7^ μm^3^/J under CW–femtosecond filament irradiation. These results demonstrate the potential of highly localized ultrashort-pulse energy deposition for achieving high normalized material removal efficiency. In contrast, the present study employs a substantially larger CW spot of 1.2 mm and a 0.5 mm ns spot, with CW power of up to 1800 W and ns pulse energies of 1–4 J. Although the normalized damage efficiency obtained in the present study is not directly comparable with these CW–fs studies because of the substantial differences in material, spot size, pulse duration, and energy-deposition mechanism, the comparison demonstrates that the present work explores a distinct large-spot CW–ns processing regime for aluminum alloy damage. In particular, the present configuration achieves a maximum damage depth of 206 μm while maintaining the transient peak temperature within approximately 3700–3800 K, indicating that the enhanced penetration is primarily associated with improved spatial–temporal energy coupling rather than simply increasing the peak temperature.

The present results are consistent with previous studies regarding the beneficial role of CW-induced thermal preconditioning, while demonstrating enhanced penetration capability under the investigated conditions. The main advancement lies in the spatial–temporal coordination of a relatively large CW thermal field and a smaller ns energy-deposition region, which provides an effective route for controlling thermal accumulation, plasma-assisted melt ejection, and transient material removal in 5052 aluminum alloy. Compared with previous CW–ns/ms studies, the present work provides a more systematic correlation among laser parameters, temperature evolution, plasma behavior, surface morphology, and damage depth, while the comparison with recent CW–fs filament studies further clarifies the distinct processing regime and application characteristics of the present CW–ns approach.

However, several limitations should be acknowledged. The plasma-shielding mechanism in the present study is mainly inferred from plasma morphology and brightness observed by high-speed imaging. Quantitative plasma diagnostics, including optical emission spectroscopy, electron density, plasma temperature, and laser absorption measurements, were not performed [64]. Therefore, the contribution of plasma shielding to laser-energy attenuation and material removal is discussed qualitatively rather than quantitatively [64,65]. In addition, although SEM observations provide evidence of remelting, molten-material redistribution, and localized defects, quantitative surface roughness, recast-layer thickness, and compositional characterization remain to be further investigated.

Future studies will combine spectroscopic plasma diagnostics, advanced surface/microstructural characterization, and numerical modeling to quantitatively clarify the coupling among laser energy deposition, plasma evolution, and molten-material dynamics [66]. In particular, fractional-order thermal models may provide a useful framework for describing the memory-dependent and non-equilibrium thermal response associated with continuous heat accumulation and transient pulse excitation [66,67]. Coupling such thermal models with plasma and molten-pool dynamics could further improve the understanding and prediction of temperature evolution, energy transport, and damage initiation during CW–ns combined irradiation [66,68]. Overall, the present work demonstrates that the principal advantage of CW–ns combined pulse irradiation lies not simply in achieving a higher transient temperature, but in establishing a controllable spatial–temporal energy-deposition pathway, in which continuous thermal preconditioning and transient pulse excitation synergistically regulate material removal in aluminum alloys.

Overall, the present study establishes a controllable spatial–temporal energy-deposition strategy in which CW-induced thermal preconditioning and transient ns pulse excitation synergistically regulate material removal, while the above limitations provide clear directions for further quantitative investigation.

## 4. Conclusions

In this work, the dynamic interaction mechanisms of an individual CW laser, an individual ns pulse laser, and a combined pulse laser acting on 5052 aluminum alloy were systematically investigated. The damage morphology, transient thermal behavior, and material removal mechanisms were analyzed using infrared thermography, high-speed imaging, and three-dimensional surface characterization. The main conclusions are summarized as follows:

(1) Under individual CW laser irradiation and individual ns pulse laser irradiation, only shallow surface damage was produced on the 5052-aluminum alloy. The CW laser mainly induced thermal melting, whereas the ns pulse laser caused localized ablation accompanied by plasma generation. In both cases, the material removal capability remained limited.

(2) Compared with either individual laser, the combined pulse laser significantly enhanced the damage depth and material removal efficiency. The continuous thermal input from the CW laser established and maintained a stable molten pool, while the transient impact of the ns laser pulses promoted molten metal ejection through plasma recoil pressure and shock effects. The synergistic energy coupling between the two lasers substantially improved the material removal performance.

(3) Under a fixed ns pulse energy of 1 J, the penetration depth exhibited an increase–decrease–increase trend with increasing CW laser power. This behavior resulted from the transition of the molten pool energy coupling mode, including thermal accumulation, energy redistribution, and the establishment of a deep-penetration melting regime. Under a fixed CW laser power of 1800 W, the penetration depth first increased and then decreased with increasing ns pulse energy, primarily due to the transition of plasma behavior from plasma-assisted material removal to plasma shielding, which reduced the effective laser energy coupled into the material at higher pulse energies.

(4) Based on the transient temperature evolution and high-speed imaging observations, the material removal process under combined pulse laser irradiation can be divided into four successive stages: preheating, molten pool formation, plasma expansion, and stable material removal. The dynamic evolution of these stages reveals that the material removal performance is governed by the synergistic interaction among continuous thermal accumulation, transient pulse impact, plasma evolution, and molten pool dynamics.

Overall, this study elucidates the dynamic damage mechanism of 5052 aluminum alloy under combined pulse laser irradiation and provides theoretical guidance for optimizing laser processing parameters and improving the machining efficiency and quality of aluminum alloys. More importantly, this work demonstrates that the material removal efficiency under combined pulse laser irradiation is fundamentally governed by the dynamic balance between plasma-assisted enhancement and plasma-shielding suppression, providing new insights into the optimization of combined laser processing of aluminum alloys.

## Figures and Tables

**Table 1:** Comparison of representative combined-laser studies with the present work.

Study	Material	Laser Configuration	Spot Size	Laser Input	Damage Performance
Jia et al. [63]	Q235B steel	CW + ns	CW: 150 μm; ns: 40 μm	CW: 100–700 W; ns: ~0.6 mJ	Drilling enhancement; recast layer up to 200 μm
Ding et al. [52]	Si, 316L	CW + pulsed laser	CW: 5 mm; ns: 22.5/40.3 μm	CW preheating + ns pulsed laser	Ablation rate increased with substrate temperature
Yuan et al. [53]	Al alloy	ms + ns	CW: 0.48/0.76 mm; ns: 1.30/0.44 mm	ns: 1.5 J; ms: ~20 J	Depth: 125.37–149.24 μm
Guo et al. [34]	Alumina ceramic	CW + fs filament	CW: 167 μm; ~30 μm filament	CW: 500 W; fs: 1 mJ	Damage efficiency: 1.51 × 10^7^ μm^3^/J
Li et al. [47]	Alumina ceramic	CW + fs filament	CW: ~140 μm; filament: ~290 μm	CW: 500–700 W; fs: 1 mJ	Ablation efficiency: 1.37 × 10^7^ μm^3^/J
Present study	5052 Al alloy	CW + ns	CW: 1.2 mm; ns: 0.5 mm	CW: 1200–1800 W; ns: 1–4 J	Maximum depth: 0.206 mm; maximum damage efficiency: 5.48 × 10^−6^ mm^3^/J

## Data Availability

The original contributions presented in the study are included in the article; further inquiries can be directed to the corresponding author.
